# Reconstruction method and optimum range of camera-shooting angle for 3D plant modeling using a multi-camera photography system

**DOI:** 10.1186/s13007-020-00658-6

**Published:** 2020-08-31

**Authors:** Xingtong Lu, Eiichi Ono, Shan Lu, Yu Zhang, Poching Teng, Mitsuko Aono, Yo Shimizu, Fumiki Hosoi, Kenji Omasa

**Affiliations:** 1 Graduate School of Agricultural and Life Sciences, The University of Tokyo, 1-1-1 Yayoi, Bunkyo, Tokyo 113-0032 Japan; 2 Faculty of Agriculture, Takasaki University of Health and Welfare, 54 Nakaorui-machi, Takasaki, Gunma 370-0033 Japan; 3 School of Geographical Sciences, Northeast Normal University, 5268 Renmin Street, Changchun, 130024 China; 4 National Institute for Environmental Studies, 16-2 Onogawa, Tsukuba, Ibaraki 305-8506 Japan; 5 Research Center for Agricultural Information Technology, National Agriculture and Food Research Organization, 3-1-1 Kannondai, Tsukuba, Ibaraki 305-8517 Japan

**Keywords:** Multi-camera photography, Optimum range of camera-shooting angle, Viewing zenith angle (VZA), SfM, 3D modeling, Plant structure

## Abstract

**Background:**

Measurement of plant structure is useful in monitoring plant conditions and understanding the responses of plants to environmental changes. 3D imaging technologies, especially the passive-SfM (Structure from Motion) algorithm combined with a multi-camera photography (MCP) system has been studied to measure plant structure due to its low-cost, close-range, and rapid image capturing ability. However, reconstruction of 3D plant models with complex structure is a time-consuming process and some systems have failed to reconstruct 3D models properly. Therefore, an MCP based SfM system was developed and an appropriate reconstruction method and optimal range of camera-shooting angles were investigated.

**Results:**

An MCP system which utilized 10 cameras and a rotary table for plant was developed. The 3D mesh model of a single leaf reconstruction using a set of images taken at each viewing zenith angle (VZA) from 12° (C2 camera) to 60° (C6 camera) by the MCP based SfM system had less undetected or unstable regions in comparison with other VZAs. The 3D mesh model of a whole plant, which merged 3D dense point cloud models built from a set of images taken at each appropriate VZA (Method 1), had high accuracy. The Method 1 error percentages for leaf area, leaf length, leaf width, stem height, and stem width are in the range of 2.6–4.4%, 0.2–2.2%, 1.0–4.9%, 1.9–2.8%, and 2.6–5.7% respectively. Also, the error of the leaf inclination angle was less than 5°. Conversely, the 3D mesh model of a whole plant built directly from a set of images taken at all appropriate VZAs (Method 2) had lower accuracy than that of Method 1. For Method 2, the error percentages of leaf area, leaf length, and leaf width are in the range of 3.1–13.3%, 0.4–3.3%, and 1.6–8.6%, respectively. It was difficult to obtain the error percentages of stem height and stem width because some information was missing in this model. In addition, the calculation time for Method 2 was 1.97 times longer computational time in comparison to Method 1.

**Conclusions:**

In this study, we determined the optimal shooting angles on the MCP based SfM system developed. We found that it is better in terms of computational time and accuracy to merge partial 3D models from images taken at each appropriate VZA, then construct complete 3D model (Method 1), rather than to construct 3D model by using images taken at all appropriate VZAs (Method 2). This is because utilization of incorporation of incomplete images to match feature points could result in reduced accuracy in 3D models and the increase in computational time for 3D model reconstruction.

## Background

During plant growth, plant structures are determined by plant genetic changes to accommodate the surrounding environment [1–4]. Measurement of the plant structures can help monitor plant conditions and increase understanding of the impact of the external environment on plants to some extent [5–9]. For example, leaf area, leaf inclination angle, and plant height reflect the biological and physical processes of vegetation, such as photosynthesis, respiration, and transpiration.

In terms of plant structure measurement, 3D imaging technologies have more advantages than 2D imaging technologies because of the complex structure of plants [6, 10–12]. Among the 3D imaging technologies, the active and passive methods are the two primary imaging techniques to measure 3D plant structure parameters quantitatively [13–16]. Light Detection and Ranging (LiDAR), one of the main active methods, has high precision as many researchers have reported [6, 17–22]. However, LiDAR has the disadvantages of being costly and time-consuming in scanning, and moreover, it is not suitable for plant reconstruction within close distance in time-of-flight monitoring [23, 24]. Also, to add color information, information from additional digital camera would be needed. RGB-D cameras, or depth cameras, are often utilized for 3D plant modeling [25]. RGB-D cameras are quite inexpensive in comparison to digital single-lens reflex (DSLR) cameras. However, image resolution of such system is lower than the common DSLR camera. The SfM (Structure from Motion) algorithm is a method for reconstructing a 3D structure from 2D image sequences [26, 27]. Active-SfM, which uses structured light for 3D reconstruction, has been used for distance measurement. Structured light is the process of projecting a known pattern (often grids or horizontal bars) onto a scene which is used to enhance plant texture information and to improve the accuracy of the calculation of the morphological parameters of a plant. Bellasio et al. [28] used a digital camera to capture a sequence of images of a kidney bean plant by rotating it and adding texture to it with coded illumination, which helps to find the feature points of the active-SfM algorithm. Nguyen et al. [29, 30] designed a multi-camera system that holds five stereo camera pairs on one arc to shoot a rotating potted plant and used structured light to enhance the plant texture information. However, the structured light may affect the natural physiological conditions of the plant and destroy the original texture and color of it, which are important in identifying the plant type and its health status.

The passive-SfM algorithm is useful for measuring the plant parameters, such as structure, leaf area, distribution of leaf inclination angle, stem height, and width, both indoors and outdoors due to its advantages of being low cost and its ability for close-range and rapid image capturing ability [23, 28, 31, 32]. Zhang et al. [33] developed a high-efficiency Multi-Camera Photography (MCP) based SfM system to measure nursery paprika plants indoors without the structured light; however, there are still some shortcomings in the research. One is that the completeness of 3D modeling is judged only by visual recognition to determine the percentage of leaf completeness subjectively. Another is that the optimum range of camera-shooting angle was not defined in the MCP based SfM system. However, if the number of the camera view angle ranges were to be increased, the processing time of the SfM algorithm would increase exponentially. Thus, selecting appropriate camera shooting angles, rather than all shooting angles, reduces the computation time without sacrificing the reconstruction accuracy.

## Methods

### The improved MCP system

**Fig. 1:**
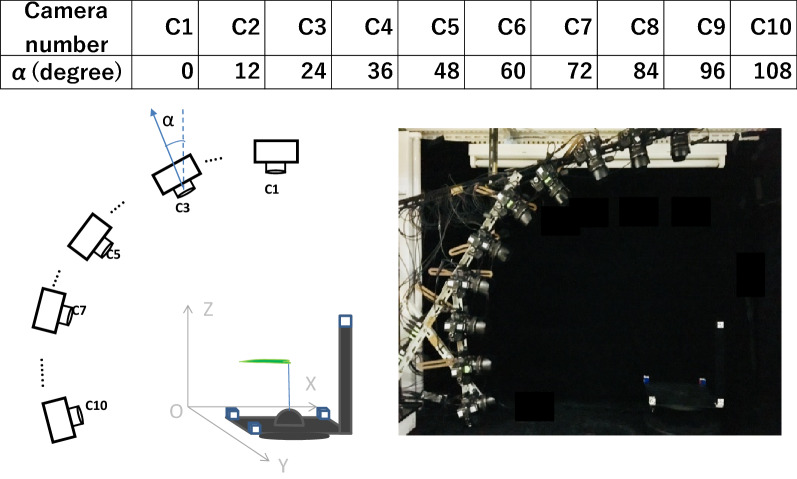
Illustration of the Multi-camera Photography system (right: the actual photograph of the system; left: schematic of the system; C1–C10 represent 10 cameras, respectively; α represents the viewing zenith angle or VZA). Two fluorescent lamps which provided constant illumination can be seen on the top and left side of the system photograph

Figure 1 shows an illustration of the MCP system used in this study. A total of 10 digital single-lens reflex (DSLR) cameras (C1 to C10) (Canon EOS Kiss X7, Canon Industrial Co., Ltd., Tokyo, Japan) was used. The camera has aspect ratio of 3:2, with resolution of 5184 × 3456 pixels which is equivalent to 18 megapixels. Effective size of a CMOS sensor is 22.3 × 14.9 mm (APS-C size). Each camera equipped with a 28 mm focal length lens (Canon EF 28 mm f/1.8 USM Lens, Canon Industrial Co., Ltd., Tokyo, Japan), were mounted on a vertical arch, equally spaced, with 12° of separation. The 28 mm focal lens is equivalent to 44.8 mm focal lens when used with an APS-C size sensor. The target plant was placed at the center of a rotary table, and each camera was placed approximately 1 m away from the target plant. The horizontal angle of view is 44°, the vertical angle of view is 30° and the diagonal angle of view is 52°. The rotary table stopped 3 s following each 2 s rotation, and each camera was remotely controlled to shoot the plant simultaneously every time the rotary table stopped. Forty-one images were acquired when the camera finished rotating 360°. A 27 cm × 27 cm black matte square plate was placed between the target plant and the rotary table. A cube with side length of 2 cm was placed on each of the three corners of the plate. Coded targets (CTs) were labeled on the outsides of each cube, and they were also placed 29 cm directly above the other corner of this plate. These CTs containing coordinate information that could be automatically detected by PhotoScan software (Agisoft LLC, St. Petersburg, Russia) were fixed on the black plate, making it convenient to construct the spatial coordinate system of the 3D model. PhotoScan software utilizes following four steps. The first step is feature matching across the photos which works similar to the well-known SIFT (Scale-Invariant Feature Transform) approach, but uses different algorithms for slightly higher alignment quality. The second step is solving for camera intrinsic and extrinsic orientation parameters. The third step is dense surface reconstruction, and the last step is texture mapping. On the top and left side of the multi-camera platform, two fluorescent lamps (FLR40S·W/M) were placed to illuminate the target plant. Photosynthetic Photon Flux Density (PPFD) on the target plant was between 10 to 12 μmol m^−2^ s^−1^. The lighting system provided moderate intensity of light from various angles. The black cover was used as background because of low reflectivity. Black background has been used in previous research [29, 33]. Camera parameters including aperture, shutter speed, ISO, and white balance were manually set to obtain the best quality image for camera calibration and stereo matching. Viewing zenith angle (VZA) was defined as the angle between the zenith and the line of sight to the camera.

## 3D model processing of one leaf

**Fig. 2:**
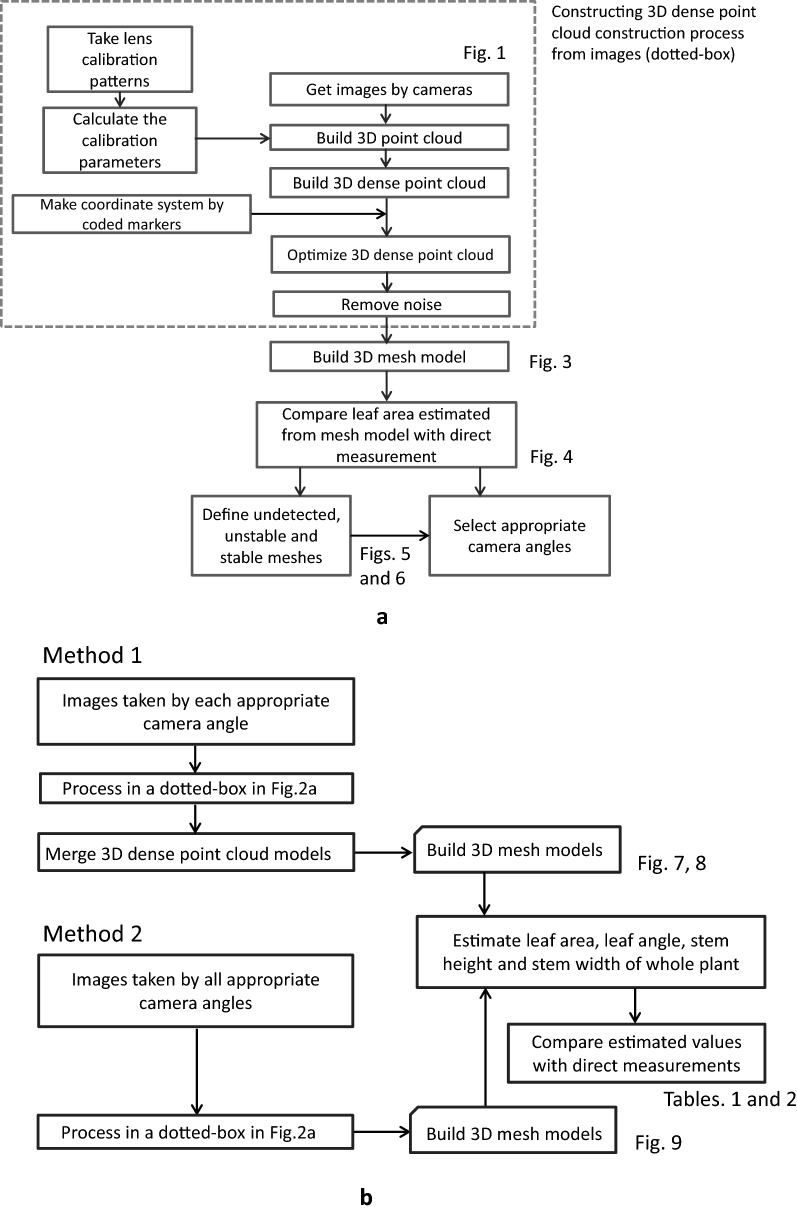
Flow chart of the 3D modeling process and exploration of the effect of camera-shooting angles to the accuracy of 3D modeling. **a** process for leaf blade, **b** processes for Method 1 and Method 2

The plant sample used for a single leaf modeling was spiny oleaster (*Elaeagnus pungens*), which is a common plant in Japan. In order to eliminate other interfering factors, such as overlapping, as well as to simply explore the effect of camera-shooting angle on leaf blade modeling, one branch of the plant with only one leaf remaining was intercepted and placed on the moist support (flower mud which can keep the branch moist) on the rotary table, which was controlled by a stepping motor. The leaf was at a naturally flat angle and fell onto an adjustable frame. The frame was regulated to ensure that the leaf sample was always at the center of the circle formed by 10 cameras. The turntable was rotated evenly, and 41 photos with 5184 × 3456 pixels in JPEG format were taken by each camera in an arc, and then the photographic processing was completed within 4 min. As shown in Fig. 2a, there were five steps from image acquisition to 3D mesh model reconstruction. Based on the SfM algorithm, a 3D point cloud was built from 41 images taken by each camera by PhotoScan considering the calibration parameters that were calculated from the lens calibration patterns and then a 3D dense point cloud was built. Afterwards, the 3D dense point cloud was optimized, and the noise was removed by the Poisson Surface Reconstruction method, [34]. Finally, the leaf blade part of the 3D dense point cloud of one leaf was transformed into a 3D mesh model. Lengths were measured using the ruler instrument of Agisoft Photoscan. Areas were measured using Matlab (The MathWorks, Inc., MA, USA) by adding mesh sizes. Also, the angles between blade base-tip connection and zenith direction were measured using Matlab. For computation, a DELL Precision T5610 (Xeon, E5-2600 v2, Dual CPUs, 96 GB RAM with a GPU of NVIDIA Quadro K600) was used for the process.

### Definition of undetected, unstable, and stable regions

In order to estimate the leaf area, the areas of all the meshes from the 3D mesh model of one leaf were summed together. Leaf area error percentage was calculated as the ratio of the difference between the estimated and the measured area of the leaf blade to the directly measured area of leaf blade as shown in Eq. (1).

1 $${\text{Leaf area error percentage}} = \frac{{\left| {{\text{Estimated area }}{-} {\text{Measured area}}} \right|}}{\text{Measured area}} \times 100\%$$

The 3D mesh model which showed the smallest error percentage was selected as the best result, and then it was used to find the optimum VZA. Generally, the area of agreement between the model and measurements were not always accurate, thus, the 3D mesh model that has the fewest undetected regions as well as the minimum difference between the estimation and direct measurements is selected as the best result. The mesh angles that occurred in this optimal model were set as the threshold to detect whether or not the regions estimated by other models are stable. Therefore, firstly, the undetected and detected regions were separated in the 3D model from each VZA angle; then the detected meshes larger than the threshold were considered unstable regions, while the meshes smaller than the threshold were regarded as stable regions.

## 3D model processing of the whole plant

Following the results of the appropriate VZAs of modeling a single leaf, a 3D mesh model of a whole flaming katy (*Kalanchoe blossfeldiana*), which is a kind of common bushy evergreen succulent plant, was developed. The leaves of this plant are also horizontally distributed broadleaves like those of spiny oleaster. The 3D reconstruction of flaming katy was used to verify the applicability of the appropriate ZAs obtained from the one-leaf experiment. When the whole plant was being modeled, some of the plant information could not be captured by only one camera which may cause the 3D mesh model to be incomplete due to leaf blade overlap. There are two ways to solve the problem of incompleteness and to improve the 3D mesh model processing of the whole plant. One is to build 3D dense point cloud models from a set of images taken at each appropriate VZA and then merge them into a new 3D dense point cloud model. Then 3D mesh model was constructed (Method 1). The other is to build a 3D mesh model of a whole plant directly from a set of all images taken at appropriate VZAs (Method 2). The detailed process was illustrated in Fig. 2b.

## 3D feature extraction of a whole plant

This section focuses on the definition of plant height, leaf length, leaf width, stem height on each part, and stem width of the 3D mesh model. Plant height is the absolute difference in the Z direction between the top of the plant and the intersection of the plant and the soil surface. Leaf length is the length of the line connecting the leaf tip and leaf base on the 3D leaf model. Leaf width is the length of the widest lobes of the leaf blade perpendicular to the line connecting the leaf tip and leaf base. Stem height for each part is the length of each internode from soil surface. Stem width is the diameter of the middle part of each internode.

Error percentage over plant height is the ratio of the difference between the estimation and the measurement to plant height as shown in Eq. (2).

2 $${\text{Error percentage over plant height}} = \frac{{\left| {{\text{Estimated value }}{-} {\text{Measured value}}} \right|}}{Plant\,height} \times 100\%$$

The ratio was calculated to make a comparison with the previous results of Nguyen [29], in which the error percentage over plant height was applied.

## Results

### 3D modeling reconstruction of one leaf blade

**Fig. 3:**
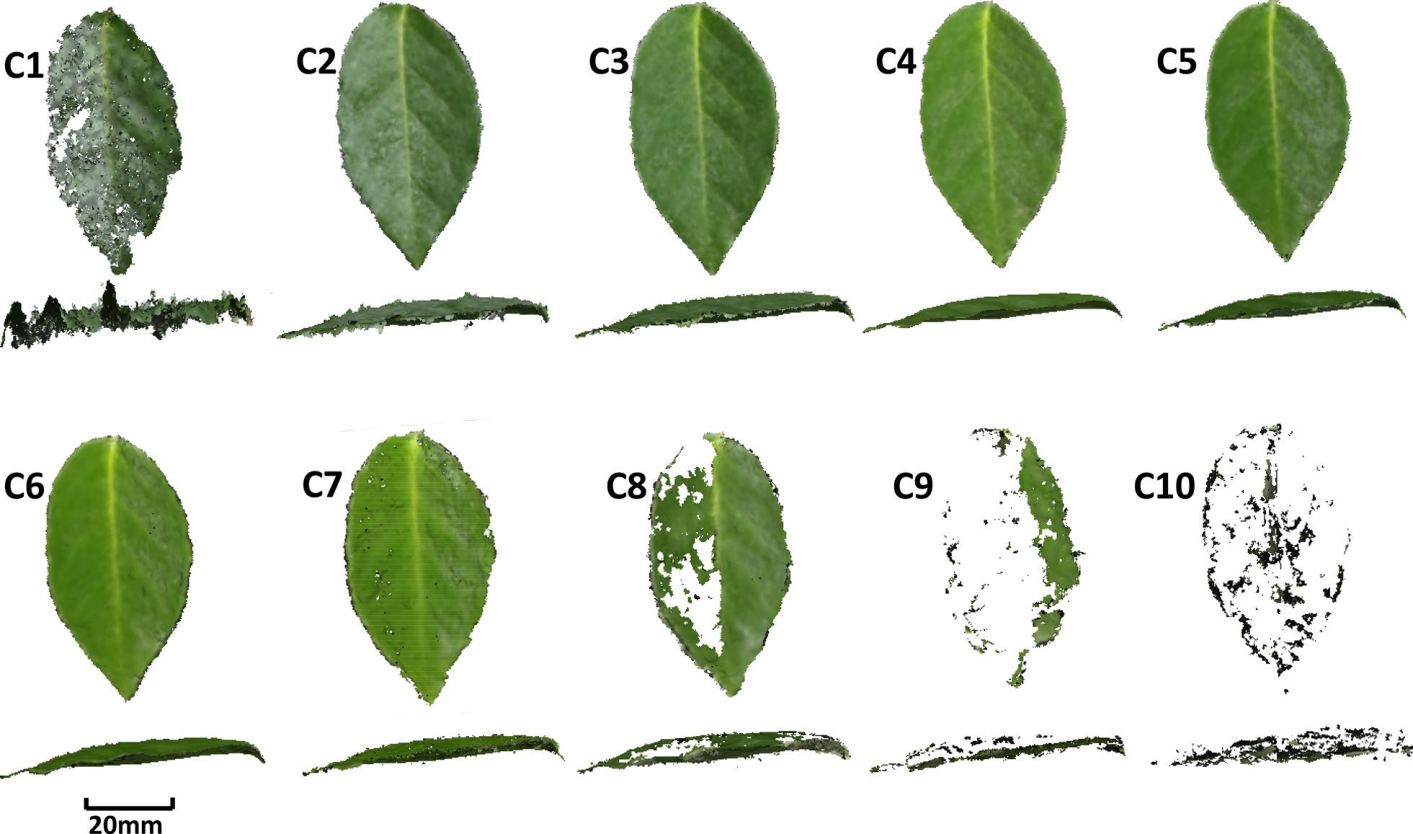
Top and side views of leaf 3D mesh models made by images taken by 10 cameras. C1 to C10 represent 10 cameras, respectively

**Fig. 4:**
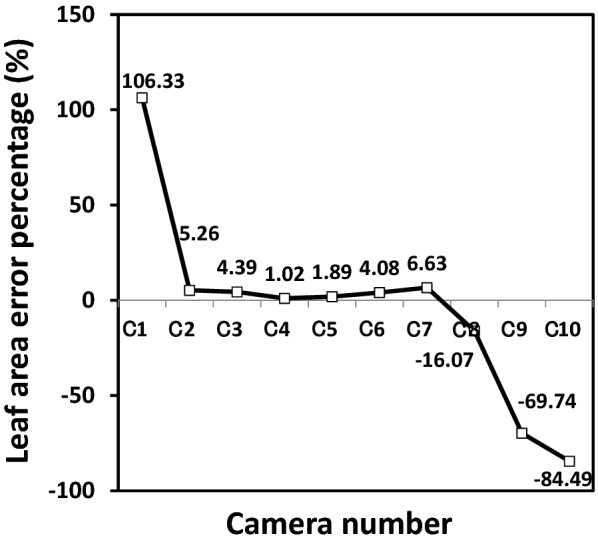
Relationship between cameras with different shooting angles and leaf area error percentage. C1 to C10 represents 10 cameras, respectively

The 3D mesh model of one leaf of spiny oleaster constructed by each individual camera is shown in Fig. 3, images provided for cameras numbered C1 thru C10. Looking at the top and side views of leaf 3D mesh models, it is evident that the quality of the model images taken by cameras at different angles differs. For example, the 3D mesh models made by C1 camera and C7 to C10 cameras missed some information, or resulted in an incomplete reconstruction of the leaf. A side view of C1 indicated that the model lacks proper depth information. Figure 4 shows a relationship between camera number (each camera number corresponds to a VZA in Fig. 1) and leaf area error percentage. The images taken by the C4 camera showed the smallest error (1.02% error in leaf area). It corresponded to the VZA of 36°. C1 showed 106.33% error, and C8 to C10 showed more than 10% error.

**Fig. 5:**
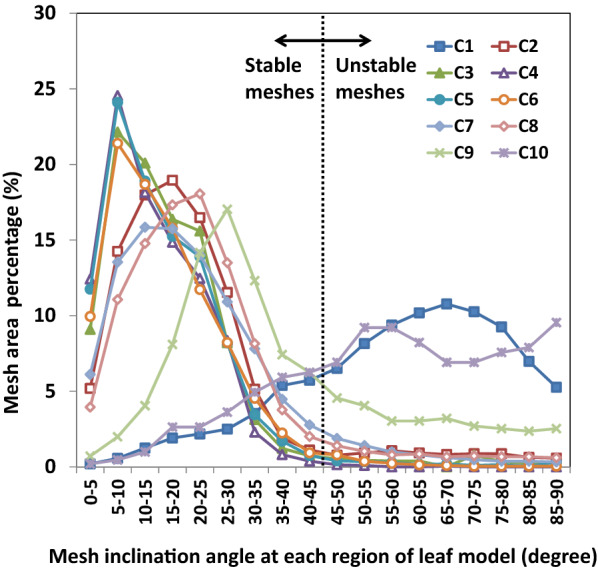
Relationship between the mesh inclination angle at each region of leaf 3D mesh models and the mesh area percentage on one leaf. Mesh inclination angle is the zenith angle of the normal vector of each mesh that makes up the model. C1 to C10 represents 10 cameras, respectively. The meshes larger than 45° in the 3D mesh model of one leaf were unstable regions, whereas those less than 45° were stable regions

A statistical analysis of the mesh inclination angle at each region on the 3D mesh model of one spiny oleaster leaf was made, as shown in Fig. 5. For the most complete 3D mesh model, made by the images taken by the C4 camera, the area of the remaining meshes was approximately 99% of all of the meshes and equal to the measured area of the leaf blade when the meshes with an inclination angle larger than 45° were removed. Thus, it was determined that the meshes larger than 45° in the 3D mesh model of one leaf were unstable regions, whereas those less than 45° were stable regions. In addition, the missing meshes were considered to be undetected regions.

**Fig. 6:**
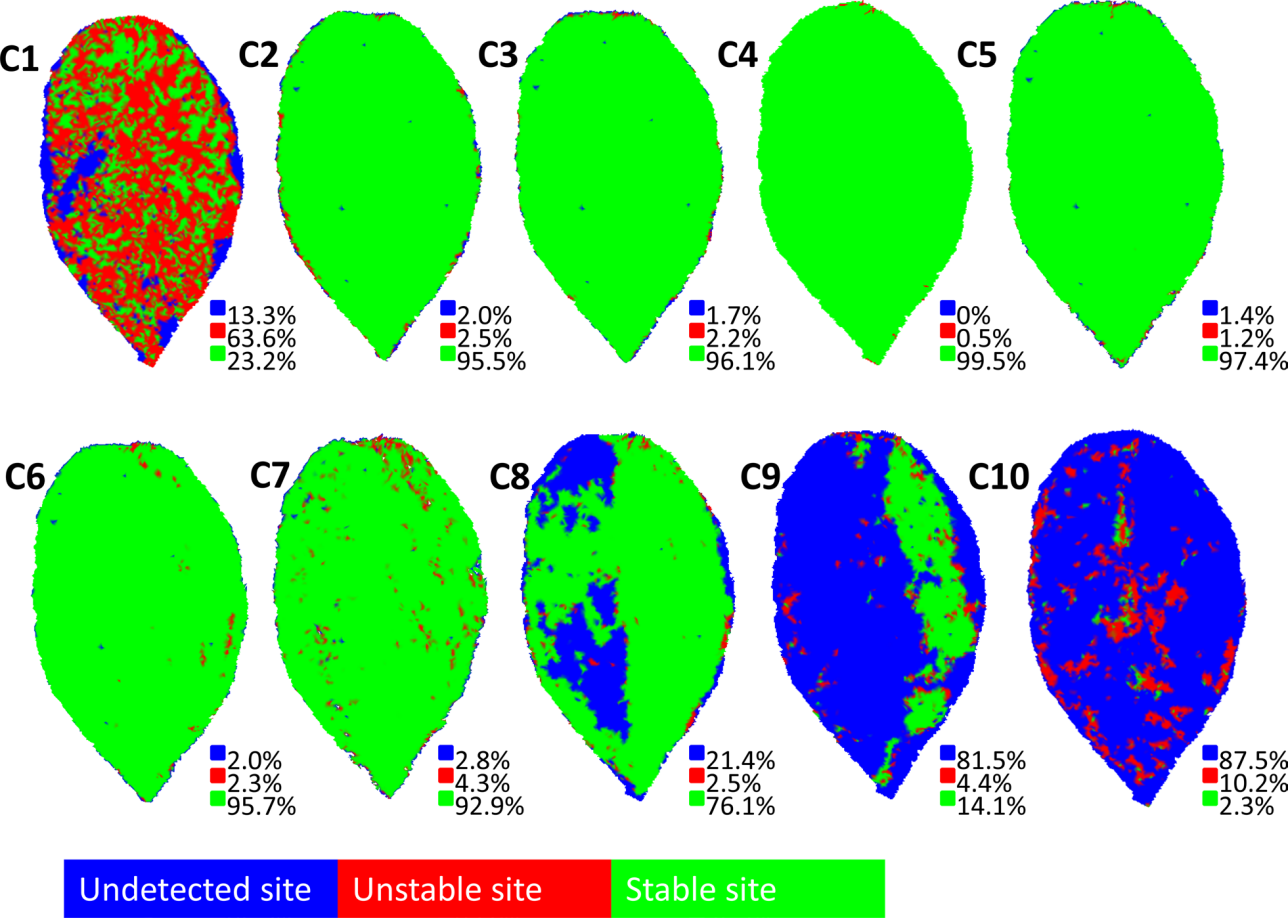
Result of the segmentation of undetected, unstable, and stable parts of leaf 3D mesh model. C1 to C10 represents 10 cameras, respectively

Figure 6 shows the undetected regions, unstable regions, and stable regions with blue, red, and green color in the 3D mesh model of one spiny oleaster leaf, respectively. The percentage of the stable areas on 3D mesh models made by images taken with cameras C2 to C6 was larger than 95%. Moreover, the percentages of the unstable areas and undetected areas on 3D mesh models made by images taken by cameras C2 to C6 were less than 2.5% and 2%, respectively. However, the stable area percentage of most of the other cameras was lower than 95%, and the percentages of the undetected areas and unstable areas of cameras C1 and C7 to C10 were larger than 2.8% and 4.3%, respectively. Considering all of these results, the angles of cameras C2 thru C6 (from 12° to 60°) were considered as the optimum.

### 3D modeling reconstruction of a whole plant

**Fig. 7:**
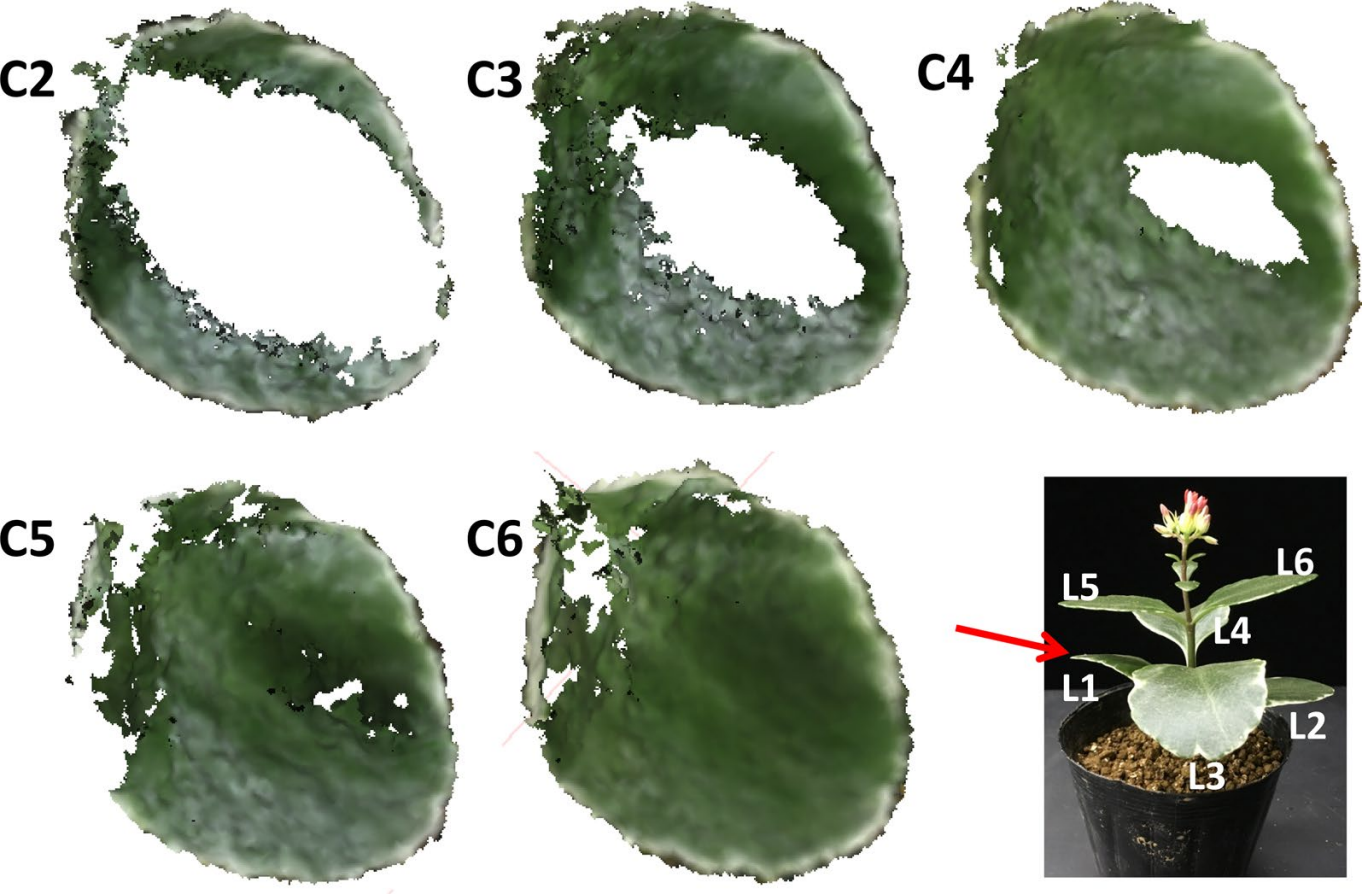
3D mesh models of leaf 1 (L1) built from images taken by C2 to C6 and 2D image of the whole plant. C2 to C6 represents cameras respectively; L1–L6 represents six leaves of the whole plant

3D mesh models of a whole plant were built from a set of images taken at each appropriate VZA between cameras C2 and C6. However, the result of the whole plant 3D mesh model was not as complete as that of the single leaf model, likely because the leaves of the whole plant were not completely visible due to the overlapping of leaves. In order to show the specific 3D mesh modeling results of the whole plant, mesh models of the first leaf (L1) of this plant were extracted from each 3D mesh model of the whole plant built from images taken at each appropriate VZA and are illustrated in Fig. 7 as examples. The 3D mesh model of L1 from camera C2 showed the lowest reconstruction rate. Only 42.6% of the area could be reconstructed in comparison to the actual area based on the direct measurement. The 3D mesh models of L1 from cameras C3 (74.2% in reconstruction rate relative to the actual area) and C4 (87.9%) were also not complete, but better than that of camera C2 with a 42.6% reconstruction rate. However, the images taken by cameras C5 (89.8%) and C6 (95.4%) could be aligned to make better 3D mesh models, although these models were also not complete and had some missing information.

**Fig. 8:**
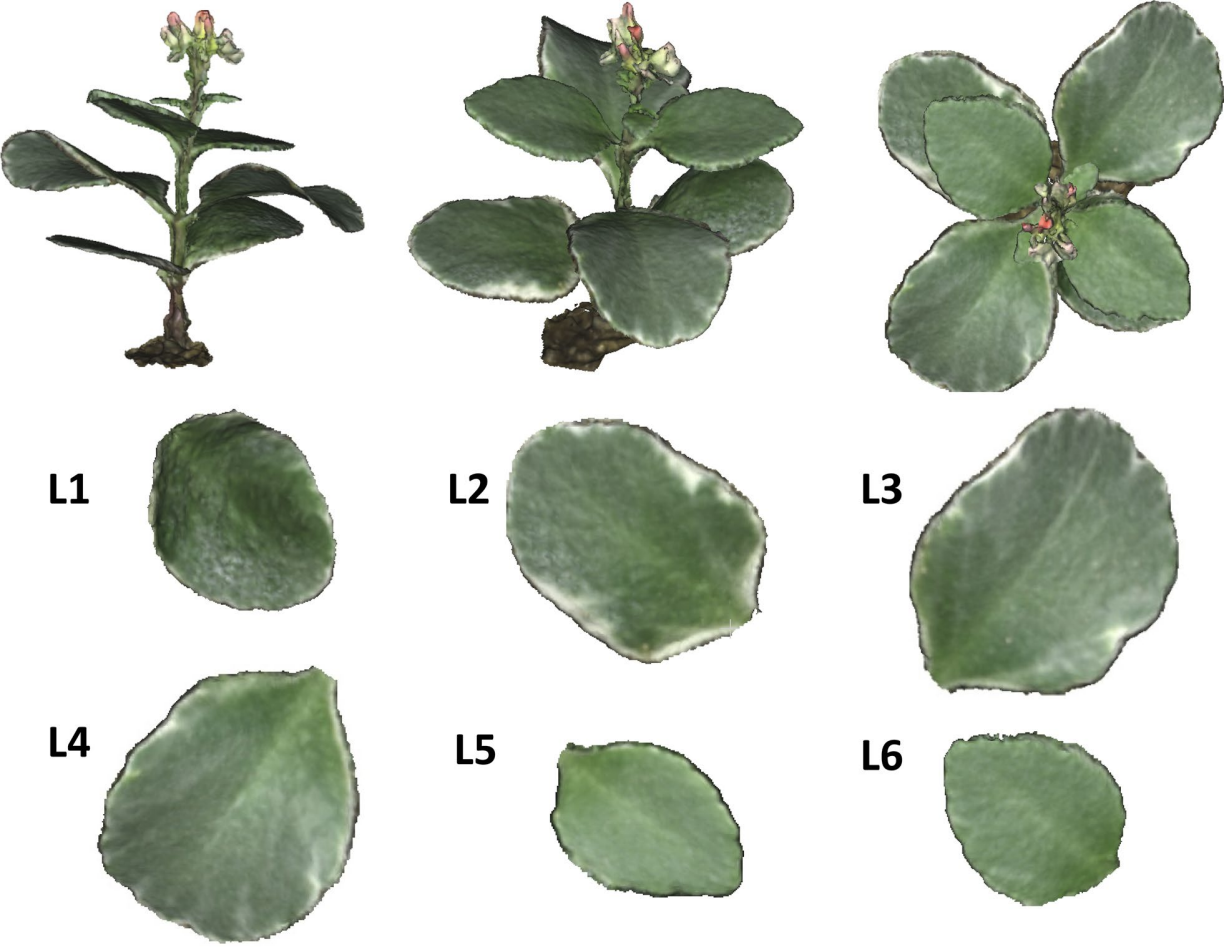
Method 1—3D mesh model of the whole plant merged by 3D mesh models built from a set of images taken at each appropriate VZA. L1 to L6 represents the 3D mesh models of six leaves, respectively

Since there was information missing on the 3D mesh models built from images taken at each appropriate VZA angle, we merged incomplete 3D dense point cloud models into a new 3D dense point cloud model. Then constructed a 3D mesh model (Method 1 in Fig. 2b), and the result is shown in Fig. 8. It can be seen that part of the stems and leaves in this 3D mesh model is better than the original model. Note that even backside of a leaf can be reconstructed (Fig. 8). It took 13 h and 41 min for the whole data processing to merge the 3D mesh models built from a set of images taken at each appropriate VZA angle.

**Fig. 9:**
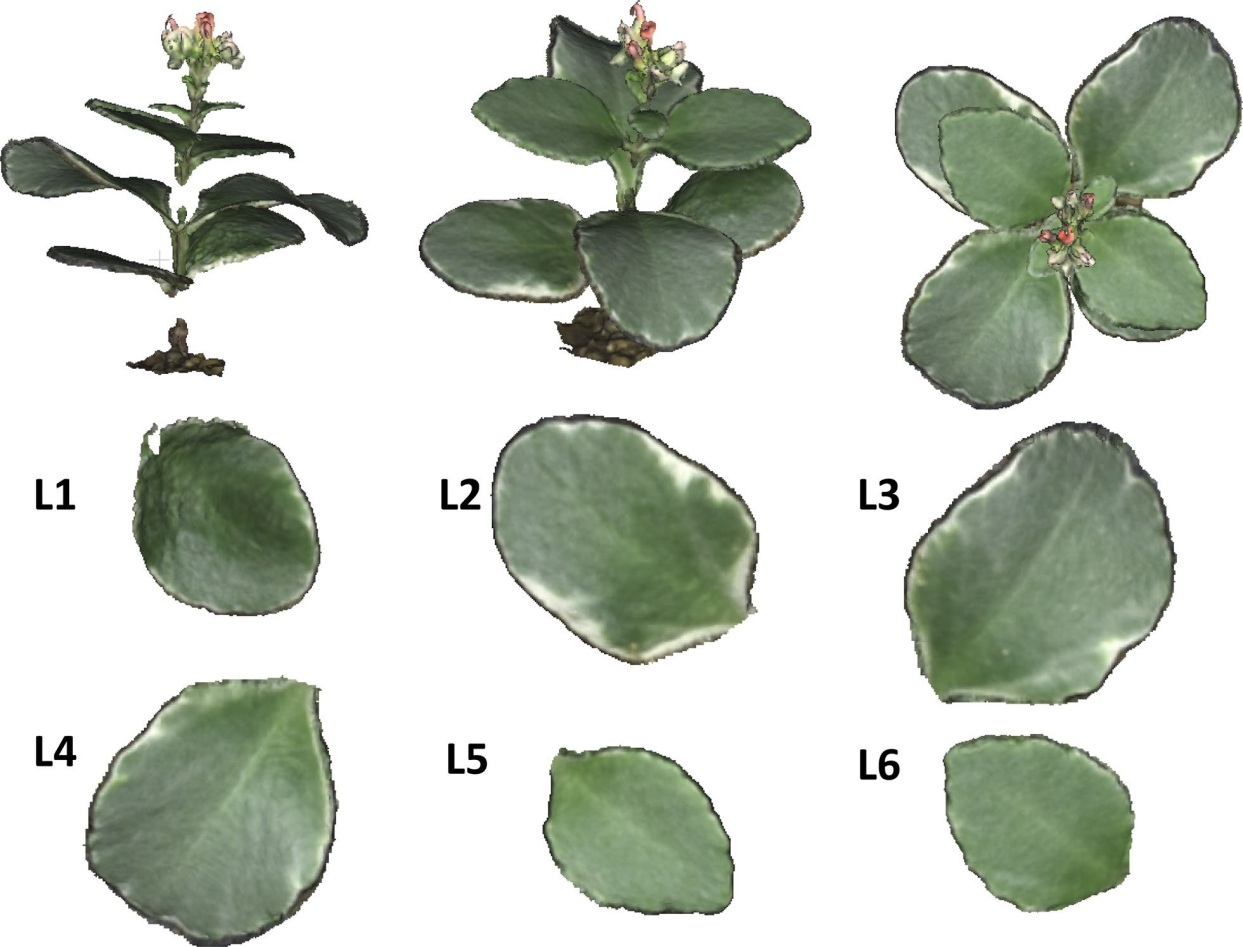
Method 2—3D mesh model built from a set of images taken at all appropriate VZAs. L1 toL6 represents the 3D mesh models of six leaves, respectively

Another method (Method 2 in Fig. 2b) to solve the problem of missing information was to build a 3D mesh model from all images taken at appropriate VZA angles from cameras C2 to C6 (Fig. 9). It can be found that the leaves of this 3D mesh model were almost complete except for L1 and part of some stems. However, the model process took 27 h, which is 1.97 times longer than Method 1.

Table 1 shows the direct measurements of leaf area, leaf length, leaf width, stem height and stem width, as well as the estimations and error percentages made for each by the two different methods. In terms of leaf estimations, the error percentages (2.6 to 4.4% for leaf area, 0.2 to 2.2% for leaf length, and 1.0 to 4.9% for leaf width) resulting from Method 1 were mostly less than those (3.1–13.3% for leaf area, 0.4–3.3% for leaf length, and 1.6–8.6% for leaf width) resulting from Method 2. At the same time, the average error percentages over plant height (98.7 mm) for leaf length (0.7%) and leaf width (1.1%) for Method 1 were also smaller than those (0.8% for leaf length, 1.5% for leaf width, respectively) for Method 2. The error percentages for stem height and width obtained by Method 1 were between 1.9 and 5.7% (the average error percentages over plant height of stem height was 0.4% and that of stem width was 0.1% for Method 1), while some of the stem error percentages could not be derived by Method 2 because some of the internodes were not detected by this method. In detail, part 1 (the internode between the soil surface and the first node) and part 3 (the internode between the second node and the third node) of the stem cannot be measured by the 3D mesh models.

**Table 1:** Direct measurement, estimation by Methods 1 and 2, and the corresponding error percentages of leaf area, leaf length, leaf width, stem height and stem width for the whole plant

	Leaf number		Direct measurement	Method 1		Method 2	
				Estimation	Error percentage (%)	Estimation	Error percentage (%)
Leaf area (cm^2^)	1		12.9	13.3	3.3	13.3	3.1
	2		14.9	15.5	4.0	15.4	3.8
	3		19.1	19.9	4.4	20.6	8.2
	4		19.0	19.5	2.6	20.7	8.9
	5		9.2	9.5	3.9	10.4	13.3
	6		10.0	10.4	4.2	10.7	7.6
	Average		14.2	14.7	3.7	15.2	7.5
Leaf length (mm)	1		49.1	49.2	0.2 (0.1)	49.4	0.6 (0.3)
	2		52.4	52.1	0.6 (0.3)	52.2	0.4 (0.2)
	3		58.2	57.1	1.9 (1.1)	57.4	1.3 (0.8)
	4		55.2	54.0	2.2 (1.2)	56.7	2.7 (1.5)
	5		42.9	42.6	0.7 (0.3)	44.3	3.3 (1.4)
	6		42.9	43.8	2.1 (0.9)	43.4	1.2 (0.5)
	Average		50.1	49.8	1.3 (0.7)	50.57	1.6 (0.8)
Leaf width (mm)	1		34.3	35.8	4.4 (1.5)	35.7	4.1 (1.4)
	2		33.8	34.7	2.7 (0.9)	36.7	8.6 (2.9)
	3		42.6	40.5	4.9 (2.1)	41.9	1.6 (0.7)
	4		44.2	43.7	1.1 (0.5)	45.0	1.8 (0.8)
	5		28.9	29.2	1.0 (0.3)	30.7	6.2 (1.8)
	6		30.0	31.3	4.3 (1.3)	31.4	4.7 (1.4)
	Average		35.6	35.9	3.1 (1.1)	36.9	4.5 (1.5)
Stem (mm)	Height	Part 1^a^	18.0	17.5	2.8 (0.5)	NG	NG
		Part 2^b^	16.0	16.3	1.9 (0.3)	15.4	3.75
		Part 3^c^	18.0	17.5	2.8 (0.5)	NG	NG
		Average	17.3	17.1	2.5 (0.4)	NG	NG
	Width	Part 1^a^	3.9	3.8	2.6 (0.1)	NG	NG
		Part 2^b^	3.5	3.3	5.7 (0.2)	3.3	5.71
		Part 3^c^	3.0	3.1	3.3 (0.1)	NG	NG
		Average	3.5	3.4	3.9 (0.1)	NG	NG

^a^The internode between the root and the first node

^b^The internode between the first node and the second node

^c^ The internode between the second node and the third node. Numbers within (brackets) are error percentages over plant height. NG: There was not enough data to construct 3D model

In addition to the leaf and stem structures, the leaf inclination angle is also an important parameter for assessing the validity of the model. Table 2 shows the direct measurement of leaf inclination angle and estimations by the two methods. A protractor was used to directly measure the inclination angle between the blade tip-base connection (base means the root of the blade) and the zenith direction (the leaf inclination angle was calculated as the complementary angle of the measured angle) in this study. It can be seen from Table 2 that the error range of the leaf inclination angle is within 5° for both methods.

**Table 2:** Direct measurement, estimation by Methods 1 and 2, respectively, and the corresponding error range of leaf inclination angle

Leaf number	Direct measurement (degree)	Method 1		Method 2	
		Estimation (degree)	Range of error (degree)	Estimation (degree)	Range of error (degree)
Leaf angle					
1	20	15	5	20	0
2	10	5	5	5	5
3	3	5	2	5	2
4	5	5	0	7	2
5	10	10	0	10	0
6	10	5	5	10	0

The leaf inclination angle was calculated as the complementary angle of the angle between the blade tip-base (root of blade) connection and zenith direction

## Discussion

### Evaluation index of the undetected, unstable, and stable regions of a model

In the previous study [28, 33, 35], the plant parameters such as leaf area, leaf length, and leaf width and the parameters of other organs estimated from 3D mesh model of plants were compared with the measurements by the coefficient of determination (R^2^) or the regression results to judge the validity of their models. Some of the studies simply subjectively judged the integrity of a plant organ by the percentage of the total number of leaves and stems of the 3D models accounting for the total number of real leaves and stems. However, in this study, a novel evaluation index of completeness of a 3D mesh model was developed. Generally, it is difficult to determine the real accuracy of the model unless the area difference between the model and direct measurement is small. Thus, the model with the fewest undetected regions as well as the smallest difference between the estimation and direct measurements is proposed to be the best leaf model. The mesh angles that occurred in this real leaf model were set as the threshold to detect whether or not the regions estimated by other models are stable. Therefore, the undetected and detected regions were separated in the 3D model from each shooting angle to select the best leaf model from all shooting angles. Then, those meshes larger than the threshold were considered unstable regions, and the meshes smaller than the threshold were regarded as stable regions. These stable and unstable regions can be used to determine the accuracy of the models. Moreover, the inclination range of both unstable and stable regions of a 3D mesh model was defined based on the relationship between the mesh inclination angle range and the actual leaf area (Fig. 5). The undetected, unstable, and stable indicators were considered comprehensively to determine the completeness of the 3D leaf mesh model.

### Optimum range of camera-shooting angles for plant modeling

The 3D mesh model created by the images taken at each VZA was statistically analyzed and displayed quantitatively in different colors (blue, undetected region; red, unstable region; green, stable region) (Fig. 6). Then we calculated the proportion of each part on these leaf models. The camera-shooting angles which provided the highest proportion of stable regions were considered optimal for modeling the plant.

According to Fig. 6, the VZA in the range of 12° to 60° was considered to be the best range. The reason for the inferior performance in the 0° to 12° VZA range was that it failed to capture the depth information of the plant model. When the VZA was more than 60°, the combined information of the adaxial surface, abaxial surface, and side of the plant was captured at the same time and resulted in a low-accuracy 3D mesh model. Because the plant was rotating, the angles between the camera-shooting angle and the inclination angle at each region of the leaf blade were always changing as the camera was shooting, thus there was no specific linear relationship between these angles and the completeness of the leaf model.

Since the structure of plants is complex, the completeness of a 3D mesh model is not only related to the camera-shooting angles, but also to the overlapping of leaves or stems and surrounding illumination. However, the influence of overlapping and illumination can be removed by aligning images from multi-views. To be specific, 3D point clouds were calculated from these images in a specified coordinate system which was able to merge a 3D model according to the points at the same condition to exclude noise points.

The appropriate background for SfM 3D reconstruction is that plant organs such as leaves and stems can be accurately separated from the background in each original 2D image set. It is also necessary that condition of light irradiation should be designed to separate the target object from the background and the object should be clearly captured. In this study, we used clear color images with a spatial resolution of 5184 × 3456 pixels which is equivalent to 18 megapixels. Although the background and light conditions may vary depending on the measurement conditions, it was relatively easy to separate the plant organs from the background in the color images, because the high-resolution color images were used. However, it might be difficult to separate the organs from the background when plant objects were in the dark shade, or when the surface of the plant showed unusual color and texture due to halation caused by specular reflection. This applies not only for indoor measurements, but also for the field measurements.

In order to obtain accurate 3D constructed images, a set of images which were measured under unfavorable conditions should be avoided. In fact, we can eliminate such data set for SfM 3D reconstruction.

In this study, an off the shelf computer was used to investigate methods, therefore long computation time was needed. However, since the optimal method (camera-shooting method and processing algorithm) were determined, this procedure can be packaged and processing time will be improved.

### Appearance of holes

There were holes in Figs. 3, 6, 7 in 3D reconstructed images. Holes appeared in Figs. 3 and 6 were due to the angle issue. Holes appeared in Fig. 7 were because of the occlusion issue. Some of small holes can be fixed by using holes filling algorithm. However, in case of large holes due to the occlusion issue, 3D reconstructions using different angles would be appropriate. Further, both methods, hole filling algorithm and utilization of images from different angles could be combined.

### Application of the whole plant modeling: comparison of two methods on leaf and stem parameters

**Fig. 10:**
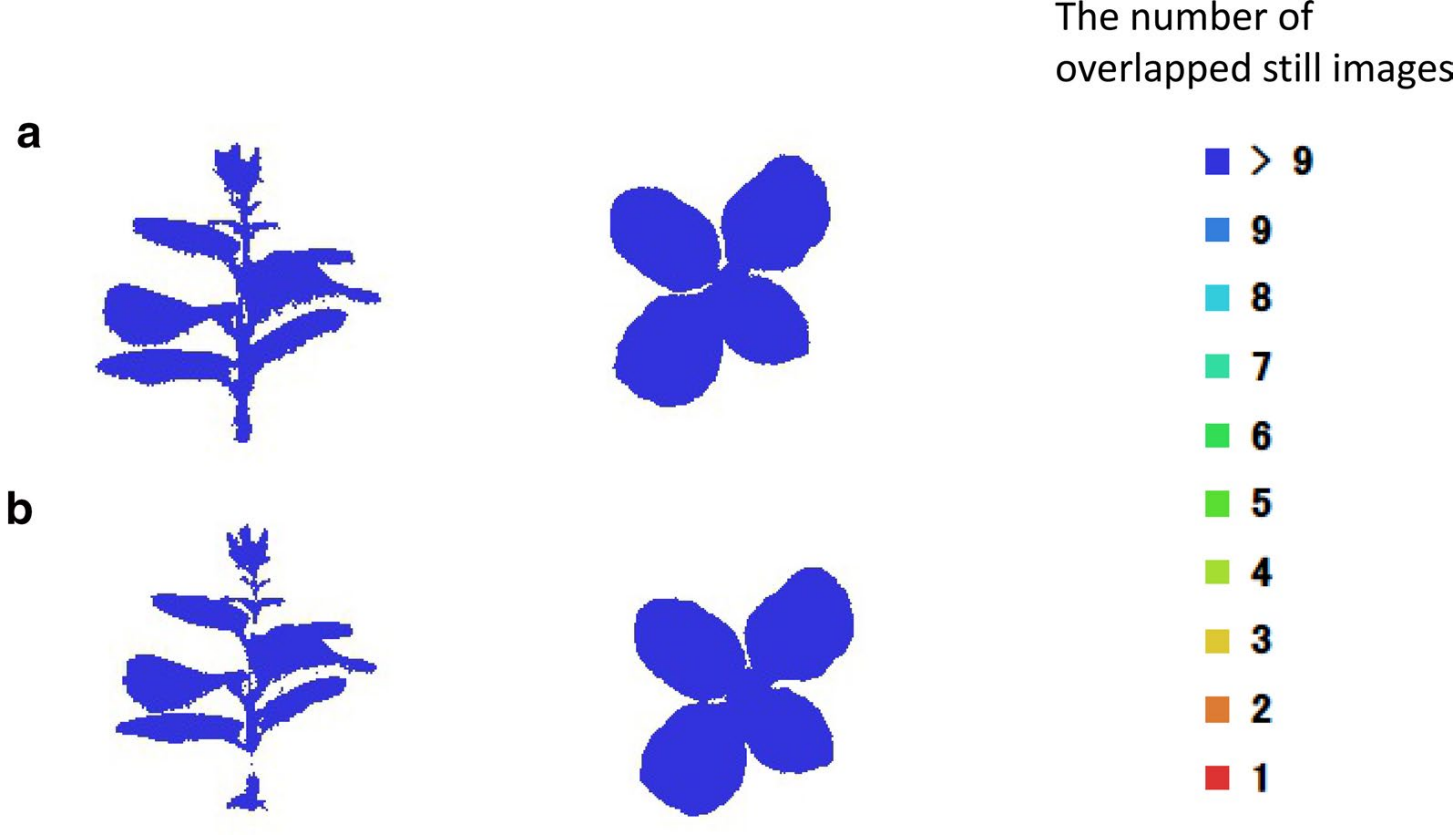
The number of overlapped still images of whole plants. **a** Method 1, **b** Method 2. Sideview in the left, and top view in the right

The method proposed from one leaf was applied to the whole plant to verify if the selected camera-shooting angles were applicable. From Tables 1 and 2, the error percentages of leaf area, length, and width from Method 1 were smaller than those of Method 2. Regarding the stem, Method 2 was also inferior to Method 1 because some information of the 3D mesh model was missing and part of the stem height and width could not be estimated. There were not enough cameras vertically to accurately extract feature points from images. This caused failure to reconstruct 3D images. This indicates that adding images with no feature points will reduce possibility of successful 3D reconstruction. The results showed that Method 1 is better in terms of computational time and accuracy. Method 1 utilizes enough number of consecutive overlapped images with sufficient feature matching points for 3D reconstruction. Then 3D dense point cloud data from different angles were merged in Method 1. In case of Method 2, images with fewer feature matching points with all angles were utilized. The estimations of leaf inclination angle obtained by the two methods were similar in that the range of error was below or equal to 5°. Therefore, it seems that there was not much difference between these two methods in terms of leaf inclination angle estimation. Figure 10 shows the number of overlapped still images of whole plants. The figure shows that Method 2 failed to reconstruct some portion of the 3D images of main stems. The time required by Method 1 for modeling was only half of that of Method 2, thus Method 1 was more efficient in time. This is caused by unnecessary images with no matching features. Therefore, Method 1 was more highly recommended when constructing a 3D model of a whole plant. Note that method 1 utilizes images taken from the appropriate angles, therefore, this method is applicable even if the appropriate angle itself is different. These facts indicate that we may apply knowledge obtained in this study to different species at different growth and developmental stages.

**Fig. 11:**
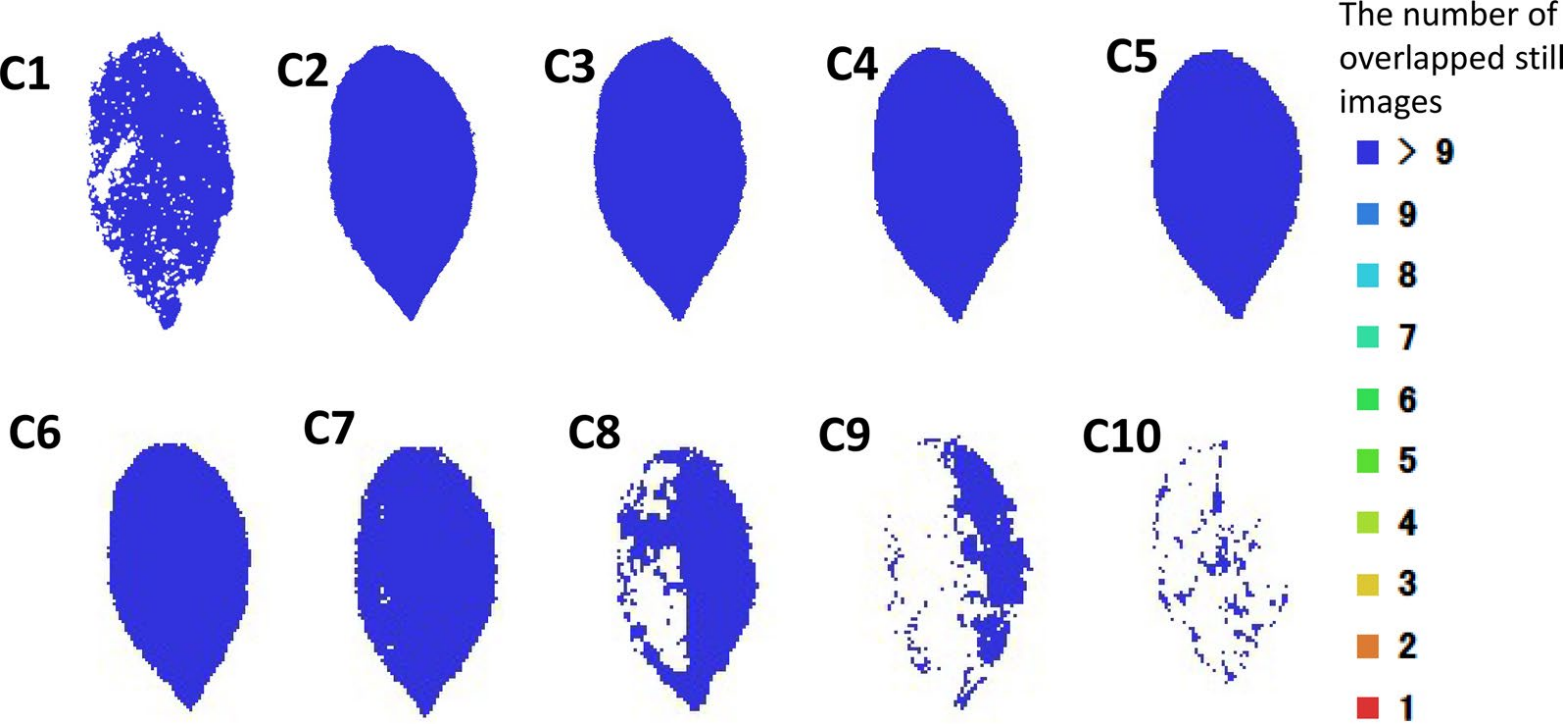
The number of overlapped still images of a leaf. C1 to C10 represents 10 cameras, respectively

Generally, an overlap of more than 90% between the adjacent images is expected in this SfM modeling algorithm. Moreover, more overlapping is needed when the modeled plant has a complex structure. In Method 1, a set of images taken at each camera-shooting angle were first applied to construct a 3D dense point cloud. Because the overlap between adjacent images in the horizontal direction was relatively larger with one picture per 9° and 41 images in a rotation, the features were easier to be captured by this processing method, which could help improve the computational efficiency of the SfM algorithm. In this case, consecutive pictures with 90% overlap and more than 9 consecutive images were needed to match adequate feature points and to reconstruct 3D images. In fact, we had enough overlap to reconstruct 3D structures for horizontal images except for C1, C8, C9, and C10 cameras as was shown in Fig. 11. But in Method 2, the overlap in the adjacent images in the vertical direction was smaller since one camera per 12° could be installed in the vertical direction. This is partially due to the size of DSLR camera and costs. This caused decrease in the number of consecutive images in vertical position, which means the features were hard to detect and points were difficult to locate accurately. Moreover, there was a significantly higher number of images required than what was needed in Method 1, thus the efficiency was worse. The interference from the upper, middle, and lower side information of a plant organ affected the accuracy of the model and also made the calculation time of the SfM algorithm longer.

Nguyen [28] used multiple pairs of stereo images taken at five different camera-shooting angles to reconstruct a 3D model of cabbage, cucumber, and tomato plants. The average error percentages over plant height of leaf length, leaf width, and internode distance were 4.87%, 3.76%, and 7.28%, respectively. However, in the present study, the average error percentages over plant height of leaf length, leaf width, and stem height on each part (equivalent to the internodes distance in Nguyen [28]) estimated from Method 1 were only 0.7%, 1.1%, and 0.4%, respectively. Thus, Method 1, which combined with the appropriate camera-shooting angles, has improved the plant parameter estimation. In terms of leaf inclination angle, the estimation values obtained by the two methods were similar either at a range of error from tip to base or average of each region of the leaf model (Table 2). The error of leaf inclination angle was less than 5°, which was stated as acceptable in a previous paper [23]. Therefore, the accuracy of the 3D mesh model of a whole plant estimated by Method 1 combined with the VZAs of 12° to 60° was assured.

Although the plant leaves used in this study were based on horizontally distributed broadleaf, the results have shown that the 3D reconstruction model has a high precision. It is expected that the distribution of nearly vertical angle leaves, such as found with wheat or corn leaves could also be modeled. Moreover, the whole plant models in this study can actually provide the leaf inclination angle information at each mesh of leaf 3D mesh models, despite that it was not used to validate the model, because it is difficult to measure the inclination angle at each mesh directly on the plant. Therefore, it is necessary to improve the method of direct measurements and the validation methods on the leaf inclination angles at every mesh in the future.

### Comparison with other 3D plant modeling systems

There are some other 3D plant modeling systems such as RGB-D camera and LiDAR. In terms of cost, to construct an MCP system, at least five sets of digital single-lens reflex camera (400 USD) and 28 mm lens (200–300 USD), a stepping motor and a turntable (100 USD), a controller computer (300 USD), a camera holding structure (500 USD), light source (50 USD) are needed. In total, it costs roughly 4450 USD. Note that resolution of inexpensive digital cameras is constantly increasing. On the other hand, RGB-D camera, which costs around 300USD, is an inexpensive option for 3D reconstruction. For example, Paulus et al. [25] showed that the data obtained commercial RGB-D system (Microsoft Kinect) can be reasonable for some parameters but not all parameters. However, image resolution of such system is around 1920 × 1080 pixels. This is equivalent to 2 mega pixels, which is lower than 18 mega pixels of a DSLR camera. Therefore, in terms of image resolution, the MCP system still has an advantage. In addition, multiple RGB-D cameras or some kind of moving system would be needed for reconstruction of 3D to capture complex images of plants. Short range LiDAR, which costs around 4000 to 5000 USD, is another option. However, LiDAR has occlusion issue. LiDAR cannot measure a target which is hiding behind some objects. For such case, multiple LiDAR systems or some platform may be needed. Also, LiDAR system only capture 3D structural information. To add color information, at least one digital camera is needed. Therefore, the MCP system has some advantages over other alternative 3D plant modeling systems under certain conditions.

The MCP based SfM system can be used for lab experiment as well as in-field experiment. 3D reconstruction of plants grown under growth chamber and laboratory are commonly conducted for plant phenotyping. Also, plant experiments and plant production under artificial lighting are increasingly important. Such measurements are conducted in the indoor conditions and can be conducted by using the system described in this article.

## Conclusions

In previous studies on multi-view measurement of plants, only the effect of the algorithm on the efficiency and accuracy of the model calculation had been considered. In contrast, this research explored the effect of camera-shooting angles on the efficiency and accuracy of the model calculations based on defining the undetected, stable, and unstable regions.

On a leaf scale, the proportion of the stable, unstable, and undetected parts of the 3D leaf model was comprehensively considered to determine a qualified leaf blade model. The VZA range of 12° to 60° (corresponding to cameras C2 to C6) used for those qualified models was considered the optimum VZA range.

Moreover, the appropriate camera-shooting angle range was verified on a whole plant by merging the images taken from each optimum shooting angle range from the single-leaf model. Method 1 (i.e., building 3D dense point cloud models from a set of images taken at each appropriate camera-shooting angle, and then merging them into a new 3D dense point cloud model. 3D mesh model which was constructed using the merged 3D dense point cloud model) was better than Method 2, as it took into consideration the error percentages for leaf area, leaf length, leaf width, stem height and stem width for each part, as well as the error range for leaf inclination angles. Also, it had the added benefit of a shorter model processing time. Further, it was confirmed that an accurate 3D mesh model of a whole plant can be obtained from images shot by an optimum range of camera-shooting angles. These foundlings will improve accuracy and time performance of MCP based SfM for measurements of plant structures. In this study, an off the shelf computer was used to investigate methods, therefore long computation times were needed. However, since the optimal method (camera-shooting method and processing algorithm) were determined., this procedure can be packaged and processing time will be improved

In summary, in this study, we determined the optimal shooting angles on the MCP based SfM system developed. We found that it is better in terms of computational time and accuracy to merge partial 3D models from images taken at each appropriate VZA, then construct complete 3D model (Method 1), rather than to construct 3D model by using all images taken at all appropriate VZAs (Method 2). This is because utilization of inappropriate VZAs and incorporation of incomplete images to match feature points could result in reduced accuracy in 3D models and the increase in computational time for 3D model reconstruction.

## Data Availability

The datasets used and/or analyzed during the current study are available from the corresponding author on request.

## Abbreviations

MCP: Multi-Camera Photography
LiDAR: Light detection and ranging
CTs: Coded targets
C: Camera
L: Leaf
SfM: Structure from Motion
VZA: Viewing zenith angle
DSLR: Digital single-lens reflex
